# Meningococci of Serogroup X Clonal Complex 181 in Refugee Camps, Italy

**DOI:** 10.3201/eid2305.161713

**Published:** 2017-05

**Authors:** Paola Stefanelli, Arianna Neri, Paola Vacca, Damiano Picicco, Laura Daprai, Giulia Mainardi, Gian Maria Rossolini, Alessandro Bartoloni, Anna Anselmo, Andrea Ciammaruconi, Antonella Fortunato, Anna Maria Palozzi, Silvia Fillo, Marino Faccini, Sabrina Senatore, Florigio Lista, Cecilia Fazio

**Affiliations:** Istituto Superiore di Sanità, Rome, Italy (P. Stefanelli, A. Neri, P. Vacca, C. Fazio);; Fondazione IRCCS Ca’ Granda Ospedale Maggiore Policlinico, Milan, Italy (D. Picicco, L. Daprai);; Agenzia di Tutela della Salute, Milan (G. Mainardi, M. Faccini, S. Senatore);; Università degli Studi di Siena, Siena, Italy, and Università degli Studi di Firenze, Florence, Italy; Careggi University Hospital, Florence (G.M. Rossolini);; Università degli Studi di Firenze, Florence (A. Bartoloni);; Centro Studi e Ricerche di Sanità e Veterinaria dell’Esercito, Rome (A. Anselmo, A. Ciammaruconi, A. Fortunato, A.M. Palozzi, S. Fillo, F. Lista)

**Keywords:** invasive meningococcal diseases, serogroup X, migrants, meningitis/encephalitis, bacteria

## Abstract

Four cases of infection with serogroup X meningococci (MenX) (1 in 2015 and 3 in 2016) occurred in migrants living in refugee camps or reception centers in Italy. All MenX isolates were identified as clonal complex 181. Our report suggests that serogroup X represents an emerging health threat for persons arriving from African countries.

Outbreaks of *Neisseria meningitidis* serogroup X meningococcal (MenX) infections in the African meningitis belt caused by isolates of clonal complex (CC) 181, including an outbreak in 2006 in Niger and one during 2007–2010 in Togo and Burkina Faso (*1**,**2*), were characterized by high rates of illness and death. Sporadic infections caused by MenX of different CCs have also been identified in Italy (*3*), Spain (*4*), and China (*5*).

Moreover, serogroup X invasive isolates from other European countries reported and available in the PubMLST database (http://pubmlst.org/neisseria/) showed high heterogeneity among themselves and with the MenX isolates of the African meningitis belt. Because of the lack of a specific herd immunity against this serogroup in Europe, non-African MenX isolates may be associated with increased host susceptibility (*4*).

Recently, the Italian Reference Laboratory for Invasive Meningococcal Disease (IMD) surveillance of the Istituto Superiore di Sanità, Rome, Italy, received samples from 4 unlinked case-patients with serogroup X IMD that occurred among migrants living in refugee camps or reception centers. The first case was reported in 2015 in a 15-year-old girl from Eritrea (ID2683) who had arrived in a refugee camp in Lombardy, Italy, 3 days before onset of disease, which manifested as septicemia. The other 3 cases were reported in 2016, two in Lombardy (in a 20-year-old man from Mali, ID2820, and a 31-year-old man from Niger, ID2849) and another in a Tuscany camp (in a 24-year-man from Bangladesh, ID2805). These cases were characterized by meningitis with fever >40°C and loss of consciousness. All patients were treated with ceftriaxone and survived. Chemoprophylaxis with rifampin or ciprofloxacin was administered to all persons directly exposed to the index case-patients. The man from Bangladesh lived in a camp with other Africa refugees for several months before disease onset, but symptoms developed in the other 3 patients shortly after their arrival in Italy.

Sample ID2849 was culture negative and characterized only by finetype (Technical Appendix Table).We performed whole-genome sequencing and assembly on the other 3 isolates by using an Illumina MiSeq sequencer (Illumina, San Diego, CA, USA) (*6*). Genomes are available through the PubMLST database, which runs on the Bacterial Isolate Genome Sequence Database platform (*7*). 

The 4 isolates were further analyzed by core genome multilocus sequence typing (cgMLST) and compared with all serogroup X genomes (n = 36) in the PubMLST database (as of December 28, 2016) (*7*). The isolates bifurcated into 2 main groups, of which CC181 genomes clustered in a single branch (Figure, panel A). Of 18 CC181 genomes, 17 were resolved in 3 main groups (Figure, panel B), according to 3 main finetypes: group 1, finetype X:P1.5–1,10–1:F1–31:ST181 (CC181); group 2, finetype X:P1.5–1,10–1:F4–23:ST5789 (CC181); group 3, finetype X:P1.5–1,10–1:F4–23:ST181 (CC181). One CC181 genome (ID LNP13407) was positioned in a branch far from the 3 main groups. Two of the strains identified in Italy in 2016 (ID2805 and ID2820) clustered in group 1 with 7 MenX strains isolated from 2005 and 2016 in Niger, Burkina Faso, Benin, and the United Kingdom (mean distance 3 loci). Notably, the UK MenX CC181 strain (ID M16_240550) clustered close to the Italy strains (mean distance 24 loci). The strain diagnosed in Italy in 2015 (ID2683) clustered in group 2 with 3 strains isolated in 2006 in Niger (mean distance 36 loci). Group 2 was strictly related to group 3, comprising 4 meningococci strains isolated during 1996–2002 in Niger. 

**Figure:**
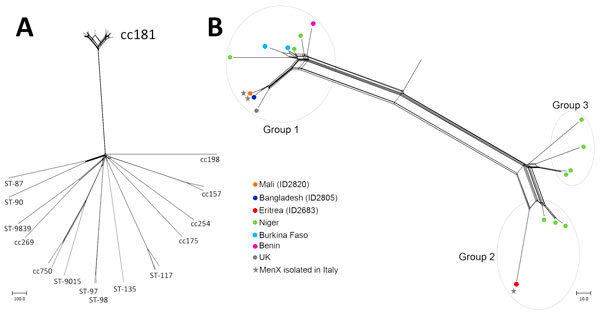
Analysis of *Neisseria meningitidis* serotype X (MenX) isolates from 3 refugees in Italy and comparison isolates from the *Neisseria* PubMLST database (http://pubmlst.org/neisseria/), as of December 28, 2016). A) Neighbor-net phylogenetic network based on a comparison of core genome loci of all MenX genomes (n = 36) available in PubMLST database. For each strain, the available designation by clonal complex (CC) or sequence type (ST) is indicated. B) Neighbor-net phylogenetic network showing 3 isolates from Italy (stars) compared with core genome loci (MenX CC181 genomes (n = 18) available in the *Neisseria* PubMLST database. Source locations for comparison isolates are indicated. Scale bars indicate number of differences among the loci compared.

As described by Agnememel et al. (*8*), MenX isolates from Africa were genetically related: they belonged to CC181and formed a single main lineage. Our genome analyses confirmed the presence of MenX strains with similar characteristics to those already described. In particular, these isolates harbored *lpt3* allele 45, previously described as a high virulence marker in the mouse model (*8*).

The analysis of meningococcal serogroup B vaccines antigens (PorA, fHbp, NadA and NHBA) identified the variants PorA VR2 10–1, fHbp-1.74 (Pfizer family B, variant B49), and NHBA-359 for samples ID2805, ID2820, and ID2849 and fHbp-1.391 (Pfizer family B) and NHBA-358 for isolate ID2683. fHbp and NHBA variant patterns had been associated with MenX CC181 isolated in Africa. NadA was absent in all analyzed MenX CC181 meningococci.

The probability of a migrant developing an infectious disease, such as IMD, after arriving in the country of destination may depend on a series of factors, such as the prevalence and incidence of the infectious diseases in the country of origin, the specific characteristics of the infectious diseases (incubation period), the number of contacts that the migrant had during the journey, and the duration of the journey. These factors should be taken into account when assessing the risk of developing specific infectious diseases, such as IMD. Our report suggests that MenX represents an emerging health threat for persons arriving in Italy from Africa. Early diagnosis, treatment, and prophylaxis should be ensured to protect vulnerable populations, including migrants, refugees, and the host community.

Technical Appendix Characteristics of meningococci of serogroup X clonal complex 181 isolated from infected patients in refugee camps, Italy.
