# Supplementary material for: Meningococci of Serogroup X Clonal Complex 181 in Refugee Camps, Italy
Source: Emerg Infect Dis. 2017 May;23(5):870–2. doi: 10.3201/eid2305.161713 (PMC5403042; doi:10.3201/eid2305.161713)
Supplement: Technical Appendix — Characteristics of meningococci of serogroup X clonal complex 181 isolated from infected patients in refugee camps, Italy. [file 16-1713-Techapp-s1.pdf]

Article DOI: http://dx.doi.org/10.3201/eid2305.161713

Meningococci of Serogroup X Clonal Complex 181 in Refugee Camps, Italy

Technical Appendix Table. Characteristics of MenX, CC181 genomes described in the analysis*

ID (ID in http://pubmlst.org/ Neisseria)	Country of origin (country of isolation)	Year	ST (CC)	PorA VR1	PorA VR2	FetA	fHbp	NHBA	*lpt3*	cgMLST group
LNP13407 (34731)	Chad	1995	ST181 (CC181)	5–1	10–1	F4–3	1.73	358	45	–
LNP14354 (34732)	Niger	1996	ST181 (CC181)	5–1	10–1	F4–3	1.391	358	136	3
97014 (34588)	Niger	1997	ST181 (CC181)	5–1	10–1	F4–3	1.391	358	136	3
98002 (34593)	Niger	1998	ST181 (CC181)	5–1	10–1	F4–3	1.391	358	136	3
2002038 (34603)	Niger	2002	ST181 (CC181)	5–1	10–1	F4–3	1.391	358	136	3
2005172 (34608)	Niger	2005	ST181 (CC181)	5–1	10–1	F1–31	1.74	359	45	1
2005166 (34740)	Niger	2005	ST181 (CC181)	5–1	10–1	F1–31	1.74	359	45	1
2006087 (34609)	Niger	2006	ST5789 (CC181)	5–1	10–1	F4–3	1.391	358	45	2
LNP23552 (34734)	Niger	2006	ST181 (CC181)	5–1	10–1	F1–31	1.74	358	45	1
LNP23557 (34735)	Niger	2006	ST5789 (CC181)	5–1	10–1	F4–3	1.391	358	45	2
2006100 (34741)	Niger	2006	ST5789 (CC181)	5–1	10–1	F4–3	1.391	358	45	2
2008223 (34612)	Burkina Faso	2007	ST181 (CC181)	5–1	10–1	F1–31	1.74	359	45	1
2008223B (34745)	Burkina Faso	2007	ST181 (CC181)	5–1	10–1	F1–31	1.74	359	45	1
2008112 (34739)	Benin	2008	ST181 (CC181)	5–1	10–1	F1–31	1.74	358	45	1
2683 (40330)	Eritrea (Italy)	2015	ST5789 (CC181)	5–1	10–1	F4–3	1.391	358	45	2
2805 (46385)	Bangladesh (Italy)	2016	ST181 (CC181)	5–1	10–1	F1–31	1.74	359	45	1
2820 (46386)	Mali (Italy)	2016	ST181 (CC181)	5–1	10–1	F1–31	1.74	359	45	1
2849	Niger (Italy)	2016	ST181 (CC181)	5–1	10–1	F1–31	1.74	359	ND	ND
M16_240550 (47293)	United Kingdom	2016	ST181 (CC181)	5–1	10–1	F1–31	1.74	359	45	1

*Men-X, meningococci serogrpup X; CC, clonal complex; ID, identification number; ST, sequence type, ND: not done;
